# Draft Genome Sequence of *Tannerella forsythia* Clinical Isolate 9610

**DOI:** 10.1128/genomeA.00024-17

**Published:** 2017-03-23

**Authors:** Sesha Hanson-Drury, Thao T. To, Quanhui Liu, Anh T. Vo, Michelle Kim, Michael Watling, Roger S. Bumgarner, Jeffrey S. McLean

**Affiliations:** a Department of Periodontics, University of Washington, Seattle, Washington, USA; b University of Washington School of Dentistry, Seattle, Washington, USA; c Department of Microbiology, University of Washington, Seattle, Washington, USA

## Abstract

We present here the draft genome sequence of *Tannerella forsythia* 9610, a clinical isolate obtained from a periodontitis patient. The genome is composed of 79 scaffolds with 82 contigs, for a length of 3,201,941 bp and a G+C of 47.3%.

## GENOME ANNOUNCEMENT

*Tannerella forsythia* is a Gram-negative, asaccharolytic, and oral anaerobe (1, 2). *T. forsythia* is a member of the “red complex,” a trio of periopathogens associated with the development of periodontal disease and inflammation (3). *T. forsythia* is strongly associated with the onset of both severe (4) and refractory periodontitis (5, 6). In comparison to its red complex fellows *Poryphyromona gingivalis* and *Treponema denticola*, little is known of the intermicrobial relationships formed and virulence mechanisms utilized by *T. forsythia* due to its fastidious growth requirements. To fully understand the role *T. forsythia* plays in the initiation and progression of periodontitis, it is vital that there is increased availability of genomic sequences. Here, we report the identification and phylogenetic placement of a draft genome sequence of *T. forsythia* clinical isolate 9610.

Isolate 9610 was collected between 1988 and 1991 at the University of Washington Graduate Periodontics Clinic from patients with various forms of periodontal disease, 5- to 10-mm pocket depths, and bleeding upon probing (*n* = 4 per patient). Samples were pooled and stored at -80°C.

In this study, 9610 was serially diluted on blood agar medium for bacterial isolation for a total of three passages, anaerobically incubated at 37°C for 3 days, then anaerobically incubated in SHI liquid medium (7) at 37°C for 3 days prior to generation of a final 20% glycerol stock solution. Genomic DNA extraction was performed utilizing the Qiagen DNeasy blood and tissue kit. The Illumina MiSeq platform was used to produce paired-end 300-bp reads, which were then assembled using SPAdes 3.9.0 (8, 9).

The draft genome consists of 79 scaffolds with 82 contigs, for a total length of 3,201,941 bp and a G+C content of 47.3%. Annotation performed by the National Center for Biotechnology Information (NCBI) Prokaryotic Genome Annotation Pipeline (PGAP) found a total of 2,620 genes, composed of 2,488 coding genes, 44 tRNAs, five rRNAs, and one clustered regularly interspaced short palindromic repeat (CRISPR).

Relatives of 9610 identified by creating a 16S rRNA gene phylogenetic tree were *T. forsythia* 8563 (10, 11), 8464 (11, 12), and Ko3 (13). When the genomic sequence of 9610 was compared to the finite available sequenced genomes of the species, composed of *T. forsythia* KS16, 92A2, and ATCC 43037, 9610 has an average amino acid identities (AAI) of 98.26%, 97.93%, and 97.77%, respectively. These results support the identification of 9610 as a novel genome to the *Tannerella forsythia* species.

When 9610 was compared to KS16, 92A2, and ATCC 43037 via Rapid Annotations using Subsystems Technology (RAST) SEED-based comparison (14), 9610 was found to possess the unique gene valine-glycine repeat protein G (VgrG). VgrG, a puncturing component (15) of the type VI secretion system (T6SS), is used by Gram-negative bacteria to deliver effectors into target cells via direct cell-cell contact (16). Further study is required to determine if 9610 possesses other distinct components of the T6SS and how the T6SS is used for microbial competition or pathogenesis.

### Accession number(s).

This whole-genome shotgun project has been deposited at DDBJ/ENA/GenBank under the accession no. MEHX00000000. The version described in this paper is version MEHX01000000.
